# Synthesis and Crystal Structure of Anhydrous Di-iodyl Carbonate (IO_2_)_2_[CO_3_], Hosting I^5+^-Cations

**DOI:** 10.1021/jacsau.5c00829

**Published:** 2025-10-14

**Authors:** Dominik Spahr, Lkhamsuren Bayarjargal, Lukas Brüning, Valentin Kovalev, Elena Bykova, Maxim Bykov, Victor Milman, Mohamed Mezouar, Björn Winkler

**Affiliations:** † 9173 Goethe University Frankfurt, Institute of Geosciences, Altenhöferallee 1, 60438 Frankfurt, Germany; ‡ Goethe University Frankfurt, Institute of Inorganic and Analytical Chemistry, Max-von-Laue-Straße 7, 60438 Frankfurt, Germany; ¶ Dassault Systèmes BIOVIA, 22 Cambridge Science Park, Cambridge CB4 0FJ, United Kingdom; § 55553 European Synchrotron Radiation Facility ESRF, 71 avenue des Martyrs, CS40220, 38043 Cedex 9 Grenoble, France

**Keywords:** di-iodyl carbonate, (IO_2_)_2_[CO_3_], synchrotron X-ray diffraction, Raman spectroscopy, density functional theory calculations, diamond anvil cell

## Abstract

The anhydrous di-iodyl carbonate (IO_2_)_2_[CO_3_] has been synthesized in a laser-heated diamond anvil cell at 30(2) GPa and 1600(200) K by a reaction of I_2_O_5_ with CO_2_. Its monoclinic crystal structure (*C*2/*c* with *Z* = 4) was determined from synchrotron single crystal X-ray diffraction data. The experimental structural model was confirmed by density functional theory based calculations in combination with experimental Raman spectroscopy. (IO_2_)_2_[CO_3_]-*C*2/*c* belongs to the family of sp^2^-carbonates, and its crystal structure is characterized by the presence of nearly trigonal-planar [CO_3_]^2–^ groups. In contrast to other iodide-containing carbonates, in this structure the iodine atoms are present as small and highly oxidized I^5+^-cations and not as large I^–^-anions. The I^5+^-cations are coordinated by seven oxygen atoms. The successful synthesis of (IO_2_)_2_[CO_3_]-*C*2/*c* represents a significant enlargement of the crystal chemistry of carbonates, as this chemically simple carbonate demonstrates that anhydrous carbonates with highly charged cations can be formed and that sp^2^-carbonates hosting a positively charged halogen atom as the only cation can be synthesized.

## Graphical abstract

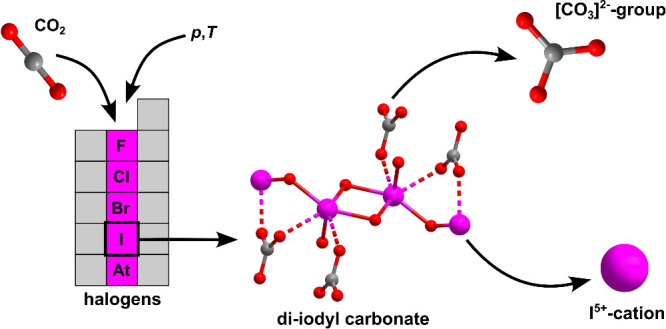

“Conventional” alkali or alkaline earth metal carbonates such as Na_2_[CO_3_] or Ca­[CO_3_] contain [CO_3_]^2–^ groups as their defining structural building blocks.^1,2^ In these trigonal-planar groups sp^2^-hybrid orbitals are present between the central carbon atom and the surrounding oxygen atoms.^3,4^ In the biosphere, the hydrosphere, soils, and the Earth’s crust, such sp^2^-carbonates are the major host of carbon. The chemically simple carbonates, i.e., carbonates containing a single type of cation for charge balancing the anionic [CO_3_]^2–^ group and no further anions, Ca­[CO_3_], (Ca,Mg)­[CO_3_]_2_, and Mg­[CO_3_] account for >90% of the carbonates present in the Earth’s crust.^4–6^ Hence, their behavior at high pressures and temperatures has been studied in great detail, and the quasi-rigid [CO_3_]^2–^ groups have been found to persist up to pressures ≥70 GPa.^7–10^ In these sp^2^-carbonates typically M^+^ or M^2+^ cations are present.

A recent significant extension of the crystal chemistry of carbonates was the discovery that chemically simple carbonates incorporating M^3+^ cations such as Fe_2_[CO_3_]_3_, Al_2_[CO_3_]_3_, and Cr_2_[CO_3_]_3_ can be synthesized from the corresponding oxide by reaction with CO_2_ in a laser-heated diamond anvil cell (LH-DAC) at moderate pressures and temperatures.^11–13^ Moreover, the recent synthesis of the low pressure phase of Be­[CO_3_], where the very small beryllium cation is in 4-fold coordination (*r*(Be^2+^) = 0.27 Å), revealed that the ionic radius of the cation is not a limiting factor for the synthesis of chemically simple carbonates.^14,15^

Halogens are very interesting as constituents from a crystal chemistry point of view as they can be incorporated either as very large anions or as small cations. For example the ionic radius of I^–^ is 2.2 Å (in 6-fold coordination), while it is only half of that value for I^5+^ (0.95 Å) and a quarter of that value for I^7+^ (0.53 Å).^15^ There are several examples of naturally occurring carbonate minerals, such as bastnaesite ((Ce,La)­[CO_3_]­F) or phosgenite (Pb_2_[CO_3_]­Cl_2_), where halogen anions occur together with the [CO_3_]^2–^-anion.^16,17^ Also, synthetic mixed-anion carbonates hosting different halogen cations (e.g., K_3_[CO_3_]­F, Ba_3_[CO_3_]­Cl_4_, Pb_2_[CO_3_]­Br_2_ or Ag_17_[CO_3_]_3_I_11_, Pr­[CO_3_]­F, Sm­[CO_3_]­F) have been obtained.^18–22^ Specifically, the recent synthesis of Na_5_(CO_3_)_2_I demonstrated that mixed-anion iodide carbonates containing I^–^-anions can be obtained at moderate pressures (18–25 GPa) in a LH-DAC.^23^

Therefore, timely and relevant questions are whether halogens can be incorporated into carbonates as cations and whether chemically simple carbonates with a single type of a pentavalent cation can be obtained. Here, we address these questions by investigating the reaction between iodine­(V)-oxide (I_2_O_5_) and CO_2_ at elevated pressures and temperatures. We adopted the synthesis approach employed for the successful synthesis of Fe_2_[CO_3_]_3_, Al_2_[CO_3_]_3_, Cr_2_[CO_3_]_3_, and Be­[CO_3_], where a corresponding oxide was reacted with CO_2_ in a LH-DAC.^11–14,24^ In I_2_O_5_ pentavalent I^5+^-cations are present. Moreover, the ionic radius of I^5+^ (*r*(I^5+^) = 0.95 Å) is similar to those often observed for the cations in “conventional” calcite-type carbonates from Ca­[CO_3_] (*r*(Ca^2+^) = 1.00 Å) to Ni­[CO_3_] (*r*(Ni^2+^) = 0.69 Å).^1,15,25^

In a first step, an I_2_O_5_ crystal was selected from a commercially available I_2_O_5_ powder, which had been dried in an oven at ≈520 K and placed on the bottom diamond of the DAC. A ruby chip for pressure determination was added.^26^ The experimental Raman spectrum of I_2_O_5_ before the cryogenic loading is in good agreement with experimental ambient pressure Raman data reported earlier.^27^ In a second step, CO_2_–I (dry ice) was added by cryogenic loading into the sample chamber of the DAC. The DAC was cooled down to ≈100 K, and the CO_2_ was directly condensed from a gas jet into the sample chamber until the chamber was filled and the I_2_O_5_ sample was completely covered. Then, the DAC was closed tightly (Figure 1a). Using Raman spectroscopy we confirmed that CO_2_–I (*Pa*3̅) is present in the sample chamber after the cryogenic loading.^28,29^

**Figure 1:**
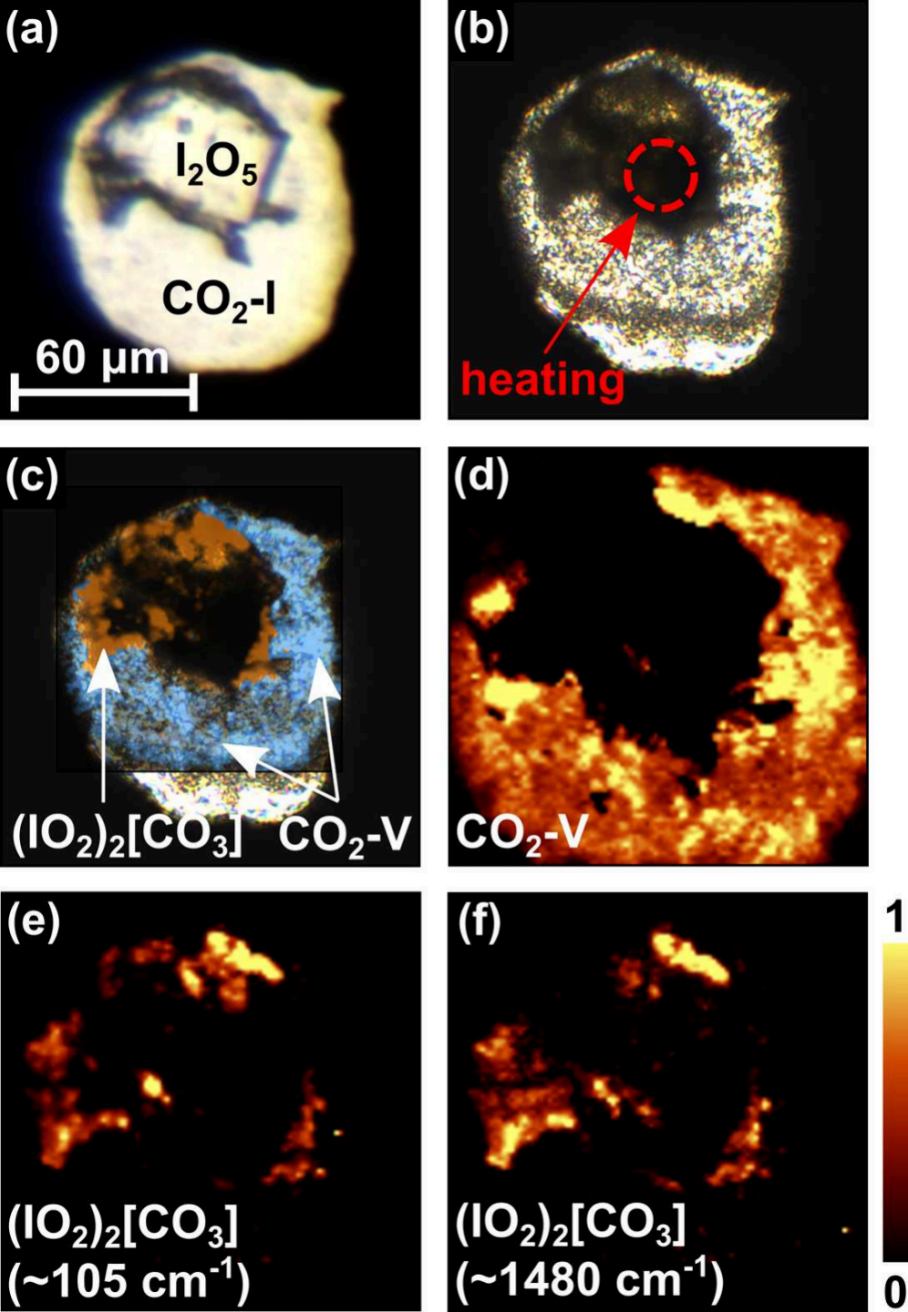
(a) I_2_O_5_ crystal in the sample chamber of the DAC together with CO_2_–I (dry ice) after the cryogenic loading. (b) I_2_O_5_ + CO_2_ mixture after laser heating to ≈1600(200) K at 30(2) GPa. (c) Combined Raman maps of (IO_2_)_2_[CO_3_] and CO_2_–V after laser heating overlaid on a picture of the sample chamber. (d) Raman map of CO_2_–V (≈900 cm^–1^). Raman maps of different (IO_2_)_2_[CO_3_] Raman modes: (e) ≈105 cm^–1^ and (f) ≈1480 cm^–1^.

The I_2_O_5_ sample in the CO_2_ was compressed to the target pressure of the experiment (≈30 GPa) without intermediate heating. In I_2_O_5_ three pressure-induced phase transitions at 5, 15, and 23 GPa have been reported in an earlier study during compression, where, during the last transition, the sample became amorphous.^27^ CO_2_ transforms from phase I to phase III (*Cmca*) in a broad (≈5 GPa) pressure range around ≈12 GPa during cold compression.^28–30^ On further cold compression, CO_2_–III remains metastable up to high pressures. Heating at pressures of ≈25 GPa causes a phase transformation to CO_2_–V (*I*4̅2*d*), which is the stable polymorph of CO_2_ up to very high pressures (>100 GPa).^30,31^ Heating CO_2_–III at pressures ≤40 GPa will lead to the formation of the high-temperature polymorphs CO_2_–II or CO_2_–IV, depending on the temperature.^30,32,33^

At 30(2) GPa the I_2_O_5_ + CO_2_ mixture was laser-heated from both sides up to a maximum temperature of ≈1600(200) K (Figure 1 b). By Raman spectroscopy we observed that the direct and indirect heating of CO_2_–III leads to the formation of CO_2_–V as the main phase, but also weak Raman bands due to the presence of CO_2_–II can be identified after laser heating (Figure 2a–c). We used spatially resolved Raman spectroscopy in order to understand if a reaction between I_2_O_5_ and CO_2_ had occurred (Figure 1c–f). We found several new Raman modes in the laser-heated area, which cannot be assigned to any of the known phases. In the center of the heated area unreacted I_2_O_5_ is still present.

**Figure 2:**
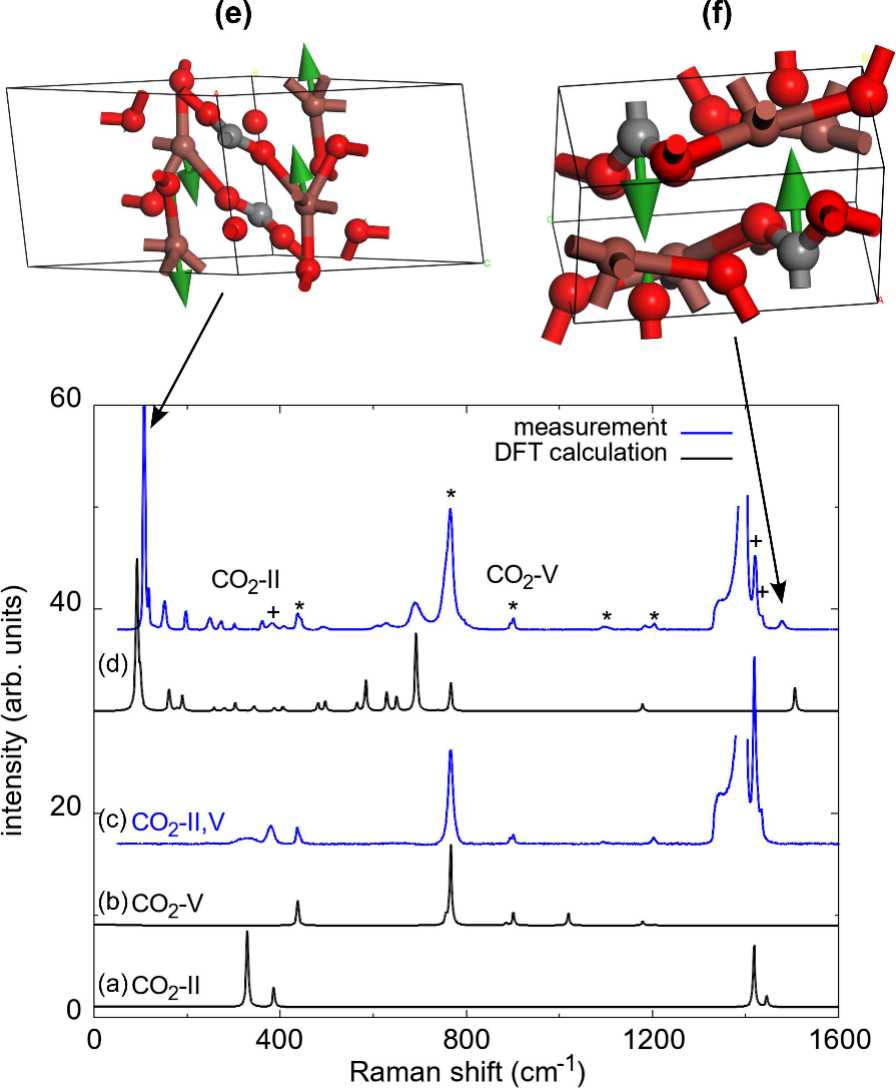
Raman spectra for (a) CO_2_–II and (b) CO_2_–V from DFT-based calculations. (c) Experimental Raman spectrum of CO_2_–V with additional peaks of CO_2_–II. (d) Experimental and DFT-calculated Raman spectra of (IO_2_)_2_[CO_3_] after the synthesis at 30(2) GPa and ≈1600(200) K. Experimental Raman spectra are shown in blue, and DFT-based calculations are shown in black. The Raman shifts of the theoretical spectra were scaled by 2–4%. Peaks of CO_2_–V are marked by an asterisk (*) and of CO_2_–II are marked by a cross (+) in the Raman spectrum of (IO_2_)_2_[CO_3_]. Eigenvector of the atomic displacements for the characteristic Raman mode (e) at ≈105 cm^–1^ and (f) at ≈1480 cm^–1^. Iodine atoms are shown in light brown, carbon atoms in gray, and oxygen atoms in red.

Hence, we employed synchrotron X-ray diffraction in order to determine the crystal structure of the unknown phase. In the first step, we measured synchrotron X-ray diffraction data on a 2D grid across the sample chamber. In the second step, we collected diffraction data suitable for single crystal X-ray diffraction on the grid positions where unidentified reflections were present. From the single crystal X-ray diffraction data we solved the crystal structure of the unknown phase (Figure 3). We found that it is a monoclinic (space group *C*2/*c*) iodine sp^2^-carbonate, (IO_2_)_2_[CO_3_]. The lattice parameters at 30(2) GPa are *a* = 14.695(5) Å, *b* = 4.429(13) Å, *c* = 5.668(14) Å, and β = 94.30(8)° (*V* = 367.9(9) Å^3^) with *Z* = 4 formula units per unit cell. The relatively low *R*_1_-value (4.9%) and the high reflection-to-parameter ratio (11:1) for a DAC experiment are indicative of a reliable structure refinement. The displacement parameters of the heavy iodine atoms were refined anisotropically, while the ones of carbon and oxygen were refined isotropically. No constraints or restraints were introduced for the structure refinement. Our DFT-based full geometry optimizations corroborated the experimental structural model. In addition, the experimental Raman spectrum is reasonably well reproduced by the one derived from our density functional perturbation theory (DFPT) calculations (Figure 2 d), confirming that the structural model is appropriate. We used a single intensity scaling factor for each of the DFT-calculated Raman spectra and one scaling factor for the Raman shifts for each of the theoretical spectra. The DFT-based geometry optimizations and Raman calculations were carried out at hydrostatic pressures, where the structure was optimized under an imposed stress tensor.

**Figure 3:**
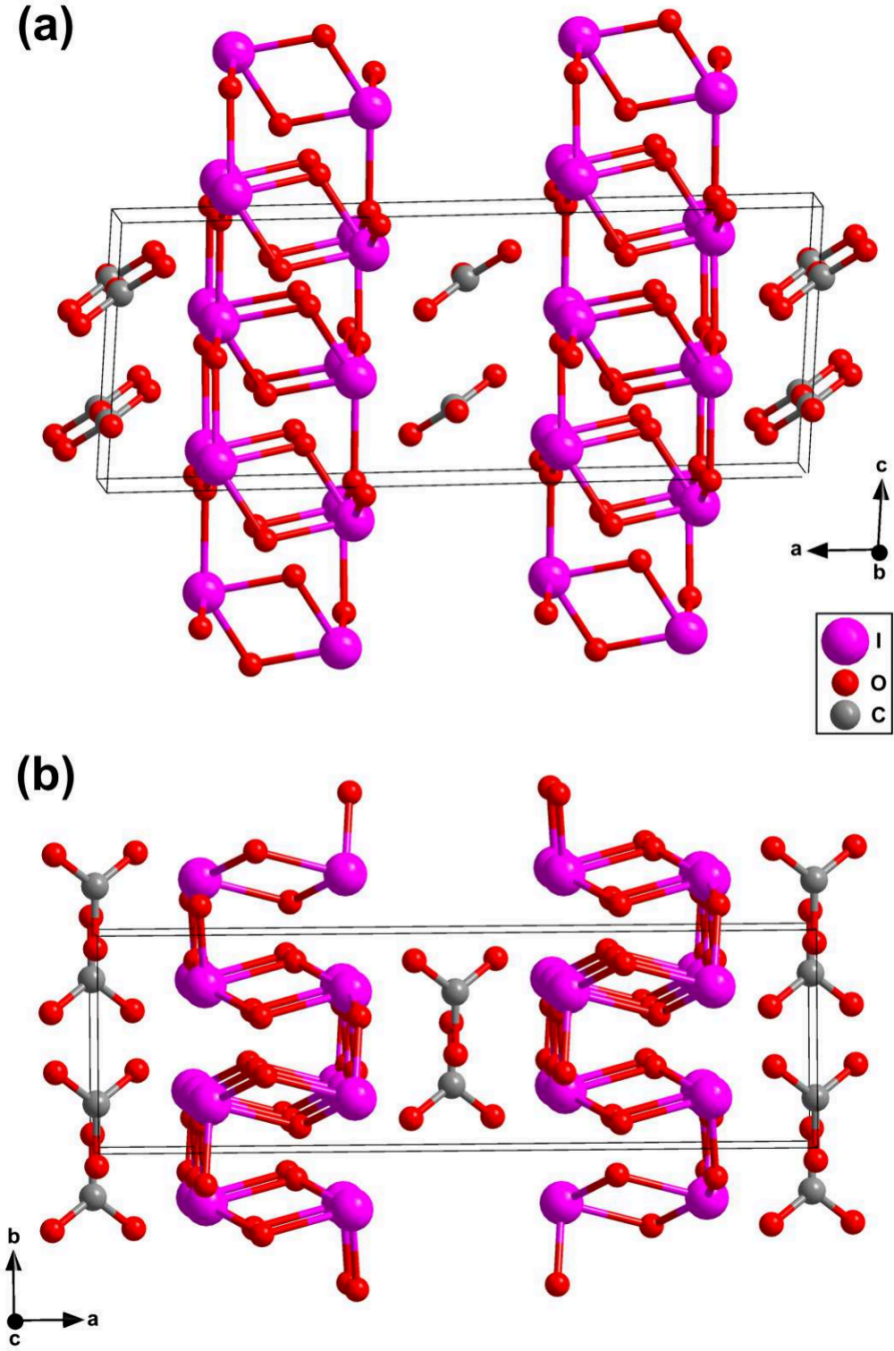
Crystal structure (*C*2/*c*, *Z* = 4) of (IO_2_)_2_[CO_3_] obtained from synchrotron single-crystal X-ray diffraction at 30(2) GPa. The crystal structure is shown (a) along the *b*-axis and (b) along the *c*-axis.

(IO_2_)_2_[CO_3_] belongs to the family of sp^2^-carbonates, characterized by the presence of nearly trigonal-planar [CO_3_]^2–^ groups (Figure 4 a). At 30 GPa, the C–O bond distances within the [CO_3_]^2–^ group are identical within the experimental uncertainties (1.25(1) Å and 1.27(2) Å) and in very good agreement with our DFT calculations (1.27 Å and 1.28 Å). The agreement between the experimental (117.0(6)° and 121.5(3)°) and the calculated (115.8° and 122.1°) O–C–O angle is a mutual confirmation of the robustness of the refinement and the reliability of the calculations, as typically [CO_3_]^2–^ groups are not that distorted. A Mulliken population analysis yielded nearly identical values for the C–O bonds (0.87 e^–^/Å^3^ and 0.85 e^–^/Å^3^).

**Figure 4:**
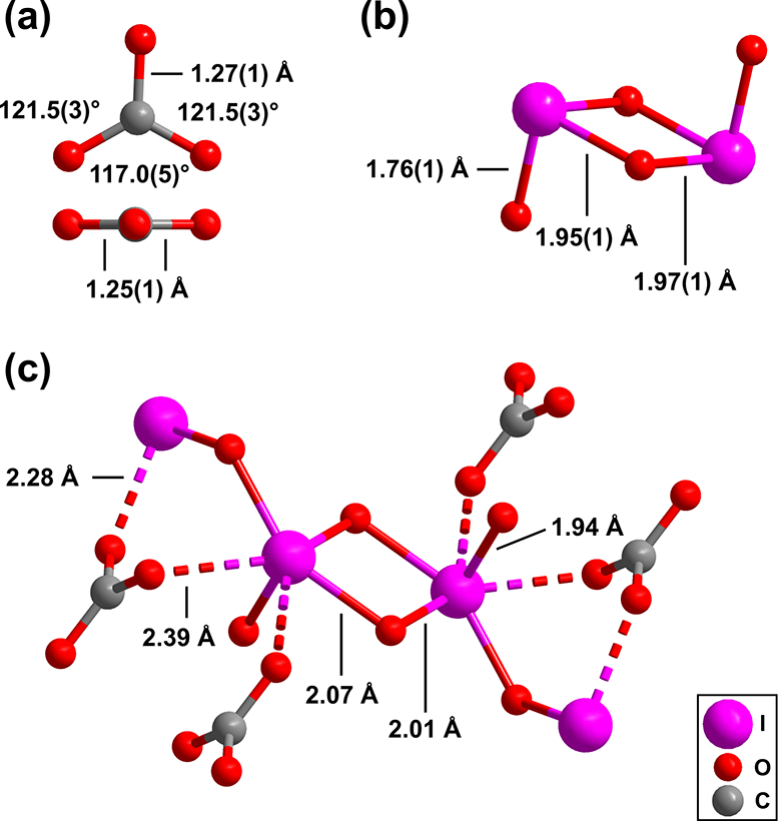
(a) Geometry of the [CO_3_]^2–^ group in (IO_2_)_2_[CO_3_] from single-crystal X-ray diffraction at 30(2) GPa. (b) Experimental geometry of the “I_2_O_4_-units”. (c) “I_2_O_4_-unit” connected to the iodine atoms of the neighboring “I_2_O_4_-units” and oxygen atoms from [CO_3_]^2–^ groups, with bond distances from DFT-based calculations.

In (IO_2_)_2_[CO_3_] the [CO_3_]^2–^ groups are arranged in layers parallel to the (302) lattice plane (Figure 3). The geometry of the [CO_3_]^2–^ groups is in good agreement with its geometry observed in other sp^2^-carbonates. In “conventional” sp^2^-carbonates such as Ca­[CO_3_]-*R*3̅*c* (calcite) or Ca­[CO_3_]-*Pmcn* (aragonite) the [CO_3_]^2–^ groups are typically trigonal-planar with O–C–O angles of ≈120° within the experimental uncertainties.^1,34^ For the two Ca­[CO_3_] polymorphs the C–O bond distances range from 1.28 Å to 1.29 Å. Nevertheless, significantly larger deviations from an ideal trigonal-planar [CO_3_]^2–^ group have been found in high-pressure carbonates such as Fe_2_[CO_3_]_3_ or Be­[CO_3_]. For example, in the crystal structure of Fe_2_[CO_3_]_3_ the O–C–O angles range from ≈111° to ≈128°, while the C–O bond distances range from 1.23 Å to 1.31 Å at 33 GPa.^11^ In Be­[CO_3_] the O–C–O angles vary between ≈117° and ≈126°, while the C–O bond distances range from 1.26 Å to 1.29 Å at 20 GPa.^14^

A second building block of the (IO_2_)_2_[CO_3_] crystal structure is “I_2_O_4_-units” (Figure 4 b). In these building blocks the I–O bond distances range from 1.76(1) Å to 1.97(1) Å at 30 GPa. The DFT-calculated I–O bond distances (1.93–2.07 Å) are up to 6% longer. This is mainly due to a bias in the refinement, which is dominated by the strongly scattering I atoms. In contrast to the strong covalent bonds within the [CO_3_]^2–^ groups, the Mulliken population ranges from 0.35 e^–^/Å^3^ to 0.06 e^–^/Å^3^ for the I–O contacts. The “I_2_O_4_-units” are connected to the next “I_2_O_4_-units” by sharing an oxygen atom, forming a network within the *b*, *c* lattice plane (Figure 3). There is only a very weak interaction with the other oxygen atoms belonging to the [CO_3_]^2–^ groups (Figure 4 c).

It is surprising that we did not observe an expected intense Raman mode in the region between 1100 cm^–1^ and 1300 cm^–1^ in the experimental Raman spectra, as such a C–O stretching mode is typical for a [CO_3_]^2–^ group in sp^2^-carbonates.^7,9^ The DFPT-calculations show that indeed there is a C–O stretching mode at ≈1130 cm^–1^, but its intensity is extremely weak in comparison to the Raman modes at lower wavenumbers (Figure 2). We used the structural model of (IO_2_)_2_[CO_3_] for DFPT calculations and calculated selected eigenvectors of the atomic displacements. The dominant Raman mode at low wavenumbers belongs to a nearly pure translation of the iodine atoms along the *c*-axis (Figure 2). In contrast, for the Raman mode at ≈1480 cm^–1^ the dominant displacement is a movement of the carbon atoms along the *b*-axis, while all other atoms remain essentially at rest, although there is a very small nonzero component in the polarization vector for the oxygen atom (Figure 2).

In order to maintain charge balance in the crystal structure of (IO_2_)_2_[CO_3_], the iodine cation has to be pentavalent (I^5+^). This is supported by our DFT-calculations. A comparison of the Mulliken charge of the iodine atoms in (IO_2_)_2_[CO_3_], which is 2.36 e^–^, to the Mulliken charge of iodine­(V) in the ambient pressure structure of I_2_O_5_, which is 2.20 e^–^, confirms that the iodine has the same valence state in these two compounds. An analysis of the electron density difference clearly shows an umbrella shaped maximum close to the iodine, which is due to a stereochemically active lone electron pair. This is the reason for the irregular coordination polyhedron around the iodine ions.

After we established that our DFT calculations accurately reproduce the experimentally determined structure of (IO_2_)_2_[CO_3_] at 30 GPa, we used further calculations to derive the *p*, *V*-relation for (IO_2_)_2_[CO_3_]. The volume contraction upon compression is shown in Figure S3, and the relative changes of the unit cell parameters are shown in Figure S4. The crystal structure of (IO_2_)_2_[CO_3_] is significantly more compressible along the *b*-axis than along the *a*- and *c*-axes. The calculated *p*, *V*-data were fitted with an equation of state (EoS) to determine the bulk modulus *K*_0_ and its pressure derivative *K*_p_. We obtained an ambient pressure bulk modulus of *K*_0_ = 15.2(6) GPa with *K*_p_ = 9.33(7). The ambient pressure bulk modulus is significantly smaller than for other “conventional” carbonates, such as Ca­[CO_3_] (67(2) GPa) or Mg­[CO_3_] (107(1) GPa), and the pressure derivative of the bulk modulus is significantly higher than those reported for other carbonates.^35^ This is often encountered in calculations when the high-pressure phase becomes unstable at lower pressures. This is the case here as well. An analysis of the elastic stiffness tensor obtained at 0 GPa shows that at this pressure the structure is unstable against small deformations. This implies that the high pressure phase is not quenchable. An analogous calculation at 10 GPa showed that at this pressure the structure is mechanically stable. Calculation of phonon dispersion curves at 30 GPa (Figure S5) showed that at this pressure, the structure is dynamically stable.

In conclusion, we have synthesized the “conventional” iodine sp^2^-carbonate (IO_2_)_2_[CO_3_] by a reaction of I_2_O_5_ and CO_2_ at 30(2) GPa and 1600(200) K. We determined the crystal structure by synchrotron single crystal X-ray diffraction and characterized the new phase by Raman spectroscopy and DFT-based calculations. The crystal structure is characterized by nearly trigonal-planar [CO_3_]^2–^ groups, which connect sheets in the *b*, *c*-plane formed by I_2_O_4_-units. The crystal structure contains pentavalent I^5+^-cations with a stereochemically active lone electron pair. Hence, the two questions posed in the introduction, namely, if a chemically simple carbonate with a halogen as a cation can be formed and if such a carbonate with a pentavalent ion can be obtained, have both been answered. This is a significant extension of the crystal chemistry of carbonates. It now is important to better understand the *p*, *T*-range in which this compound can be obtained and to determine its stability field. Moreover, we are confident that the synthesis of (IO_2_)_2_[CO_3_] provides the roadmap for the synthesis of further novel chemically simple carbonates, containing halogen atoms as cations.

## Supplementary Material



## Notes

Experimental and computational details of the experiments and the results of the single-crystal structure solution and DFT-based calculations (PDF)

## References

[1] Effenberger H., Mereiter K., Zemann J. (1981). Crystal structure refinements of
magnesite, calcite, rhodochrosite, siderite, smithonite, and dolomite,
with discussion of some aspects of the stereochemistry of calcite
type carbonates. Z. Kristallogr..

[2] Dusek M., Chapuis G., Meyer M., Petricek V. (2003). Sodium carbonate revisited. Acta
Crystallogr..

[3] Winkler B., Zemann J., Milman V. (2000). Aplanarity of CO_3_ groups:
A theoretical investigation. Acta. Cryst. B.

[4] Reeder, R. J. , Ed. Carbonates: Mineralogy and Chemistry; De Gruyter: Berlin, Boston, 1983. 10.1515/9781501508134.

[5] McKenzie N. R., Horton B. K., Loomis S. E., Stockli D. F., Planavsky N. J., Lee C.-T. A. (2016). Continental arc volcanism as the principal driver of
icehouse-greenhouse variability. Science.

[6] Hirschmann M. M. (2018). Comparative
deep Earth volatile cycles: The case for C recycling from exosphere/mantle
fractionation of major (H_2_O, C, N) volatiles and from H_2_O/Ce, CO_2_/Ba, and CO_2_/Nb exosphere ratios. EPSL.

[7] Bayarjargal L., Fruhner C.-J., Schrodt N., Winkler B. (2018). CaCO_3_ phase
diagram studied with Raman spectroscopy at pressures up to 50 GPa
and high temperatures and DFT modeling. Phys.
Earth Planet. Inter..

[8] Binck J., Chariton S., Stekiel M., Bayarjargal L., Morgenroth W., Milman V., Dubrovinsky L., Winkler B. (2020). High-pressure, high-temperature phase stability of
iron-poor dolomite and the structures of dolomite-IIIc and dolomite-V. Phys. Earth Planet. Inter..

[9] Binck J., Bayarjargal L., Lobanov S. S., Morgenroth W., Luchitskaia R., Pickard C. J., Milman V., Refson K., Jochym D. B., Byrne P., Winkler B. (2020). Phase stabilities of
MgCO_3_ and MgCO_3_-II studied by Raman spectroscopy,
X-ray diffraction, and density functional theory calculations. Phys. Rev. Mater..

[10] Cerantola V. (2017). Stability of iron-bearing carbonates in the deep Earth’s interior. Nat. Commun..

[11] Bayarjargal L., Spahr D., Bykova E., Wang Y., Giordano N., Milman V., Winkler B. (2024). High-Pressure
Synthesis of an Iron
Carbonate, Fe_2_[CO_3_]_3_. Inorg. Chem..

[12] Bayarjargal L., Spahr D., Milman V., Marquardt J., Giordano N., Winkler B. (2023). Anhydrous aluminium carbonates and
isostructural compounds. Inorg. Chem..

[13] Wang Y., Bayarjargal L., Bykov M., Bykova E., Spahr D., Glazyrin K., Milman V., Winkler B. (2025). Cr^3+^-Containing
Carbonates and Cr_2_O_3_-*Pbcn* at
Extreme Conditions. Inorg. Chem..

[14] Spahr D., Bayarjargal L., Bykova E., Bykov M., Reuter T. H., Brüning L., Jurzick P. L., Wedek L., Milman V., Wehinger B., Winkler B. (2024). Synthesis and crystal structure of
acentric anhydrous beryllium carbonate Be­(CO_3_). Chem. Commun..

[15] Shannon R. D. (1976). Revised
effective ionic radii and systematic studies of interatomic distances
in halides and chalcogenides. Acta Crystallogr..

[16] Oftedal I. (1931). Zur Kristallstruktur
von Bastnäsit, (Ce,La−)­FCO_3_. Z. Kristallogr..

[17] Giuseppetti G., Tadini C. (1974). Reexamination of the
crystal structure of phosgenite,
Pb_2_Cl_2_(CO_3_). Tschermaks Min. Petr. Mitt..

[18] Arlt J., Jansen M. (1990). Einkristallzüchtung und Röntgenstrukturanalyse
der Fluoridcarbonate K_3_CO_3_F und Rb_3_CO_3_F. Z. Naturforsch..

[19] Leyva-Bailen P., Vaqueiro P., Powell A. V. (2009). Ionothermal
synthesis of the mixed-anion
material Ba_3_Cl_4_CO_3_. J. Solid State Chem..

[20] Al’-Ama A. G., Belokoneva E. L., Dimitrova O. V., Kurazhkovskaya V. S., Mochenova N. N. (2006). Bromophosgenite: Synthesis and Structure
Solution. Russ. J. Inorg. Chem..

[21] Watanabe Y., Suzuki R., Kato K., Yamane H., Kitaura M., Ina T., Uchida K., Matsushima Y. (2021). Superionic Ag^+^ Conductor
Ag_17_(CO_3_)_3_I_11_. Inorg. Chem..

[22] Buyer C., Schumacher S. A., Schleid T. (2022). Synthesis, crystal-structure
refinement
and properties of bastnaesite-type PrF­[CO_3_], SmF­[CO_3_] and EuF­[CO_3_]. Z. Kristallogr..

[23] Yin Y., Dubrovinsky L., Aslandukov A., Aslandukova A., Akbar F. I., Zhou W., Hanfland M., Abrikosov I., Dubrovinskaia N. (2024). High-Pressure
Synthesis of the Iodide Carbonate Na_5_(CO_3_)_2_I. Solids.

[24] Spahr D., Bayarjargal L., Bykova E., Bykov M., Brüning L., Kovalev V., Milman V., Wright J., Winkler B. (2024). 6-Fold-Coordinated
Beryllium in Calcite-Type Be BeCO_3_]. Inorg. Chem..

[25] Graf D. L. (1961). Crystallographic
tables for the rhombohedral carbonates. Am.
Mineral..

[26] Mao H. K., Xu J., Bell P. M. (1986). Calibration
of the ruby pressure gauge to 800 kbar
under quasi-hydrostatic conditions. J. Geophys.
Res..

[27] Kim M., Yoo C.-S. (2016). Phase transitions
in I_2_O_5_ at
high pressures: Raman and X-ray diffraction studies. Chem. Phys. Lett..

[28] Aoki K., Yamawaki H., Sakashita M., Gotoh Y., Takemura K. (1994). Crystal Structure
of the High-Pressure Phase of Solid CO_2_. Science.

[29] Olijnyk H., Jephcoat A. P. (1998). Vibrational studies on CO_2_ up to 40 GPa
by Raman spectroscopy at room temperature. Phys.
Rev. B.

[30] Scelta D., Dziubek K. F., Ende M., Miletich R., Mezouar M., Garbarino G., Bini R. (2021). Extending the Stability Field of
Polymeric Carbon Dioxide Phase V beyond the Earth’s Geotherm. Phys. Rev. Lett..

[31] Datchi F., Mallick B., Salamat A., Ninet S. (2012). Structure of Polymeric
Carbon Dioxide CO_2_-V. Phys. Rev.
Lett..

[32] Yoo C. S., Kohlmann H., Cynn H., Nicol M. F., Iota V., LeBihan T. (2002). Crystal structure of
pseudo-six-fold carbon dioxide
phase II at high pressures and temperatures. Phys. Rev. B.

[33] Datchi F., Giordano F. M., Munsch P., Saitta A. M. (2009). Structure of Carbon
Dioxide Phase IV: Breakdown of the Intermediate Bonding State Scenario. Phys. Rev. Lett..

[34] Antao S. M., Hassan I. (2009). The orthorhombic structure of CaCO_3_, SrCO_3_, PbCO_3_ and BaCO_3_: Linear structural
trends. Can. Mineral..

[35] Zhang J., Reeder R. J. (1999). Comparative compressibilities
of calcite-structure
carbonates: Deviations from empirical relations. Am. Mineral..

