# Supplementary material for: Synthesis and Crystal Structure of Anhydrous Di-iodyl Carbonate (IO2)2[CO3], Hosting I5+-Cations
Source: JACS Au. 2025 Oct 14;5(10):4675–80. doi: 10.1021/jacsau.5c00829 (PMC12569702; doi:10.1021/jacsau.5c00829)
Supplement: Supplementary file 1 [file au5c00829_si_001.pdf]

Supplementary material: Synthesis and crystal structure of anhydrous di-iodyl carbonate $(IO_2)_2[CO_3]$, hosting I^{5+}-cations

Dominik Spahr*,a, Lkhamsuren Bayarjargal[a], Lukas Brüning[b], Valentin Kovalev[a], Elena Bykova[a], Maxim Bykov[b], Victor Milman[c], Mohamed Mezouar[d], Björn Winkler[a]

[a]Goethe University Frankfurt, Institute of Geosciences, Altenhöferallee 1, 60438 Frankfurt, Germany
[b]Goethe University Frankfurt, Institute of Inorganic and Analytical Chemistry, Max-von-Laue-Straße 7, 60438 Frankfurt, Germany
[c]Dassault Systèmes BIOVIA, 334 Cambridge Science Park, Cambridge CB4 0WN, United Kingdom
[d]European Synchrotron Radiation Facility ESRF, 71 avenue des Martyrs, CS40220, 38043 Grenoble Cedex 9, France

1. Methods

1.1. Sample material

We used commercial iodpentoxid (I_2O_5) powder (99.99% purity, Merck KGaA, Darmstadt, Germany) for our high-pressure experiments. The I_2O_5 powder was dried at $\approx$ 520 K in a drying oven for 12 h prior the loading of the diamond anvil cell (DAC). No further purification of the starting material was performed. Crystals with 30–60 µm size edge length and 10–20 µm thickness where selected for the loading of the DACs. The CO_2 gas for the gas-jet was used as purchased (Nippon gases, purity $\geq$ 99.996%).

1.2. High-pressure experiments

The high-pressure experiments were carried out in Boehler-Almax type diamond anvil cells (DACs).[1] We used diamonds with 70° opening angle on both sides and 350 µm culet size. The Re-gaskets, which were pre-indented to a thickness of $\approx$ 40 µm, and gasket holes with $\approx$ 100 µm diameter were drilled with a custom-built laser set-up. Afterward a I_2O_5 crystal with dimensions of $\approx 50 \times 30$ µm^2 and a thickness of 10–20 µm was placed on the culet of the bottom diamond. In addition, a ruby chip for pressure determination was added into the sample chamber. The pressure was determined by measuring the shift of the ruby fluorescence and we assume an error of 6% due to non-hydrostatic conditions.[2]

1.3. Cryogenic loading

After the loading of the sample the DAC was nearly closed and only a narrow gap was kept between the upper diamond and the gasket. Afterwards. CO_2-I (dry ice) was added into the sample chamber by cryogenic loading. We used a custom-built cryogenic loading system (Fig. 1). Our cryogenic loading system is an upgraded version of our prior cryo-loader employed in earlier experiments for CO_2-loading.[3] For the cryogenic loading the closed DAC containing the I_2O_5 crystal and the ruby was placed on a liquid nitrogen cooled Cu-holder and liquid nitrogen was added into the storage tank. The DAC was cooled down to $\approx$ 120 K and CO_2-I (dry ice) was directly condensed into the gasket hole from the CO_2 gas jet. We used a small nozzle to align the CO_2 gas jet with 5 l min^{-1} directly on the gap between upper diamond and the gasket. We used argon (5 l min^{-1}) as a purge gas. During the loading process the precipitation of the CO_2 in the sample chamber was monitored using an optical microscope and equipped with a camera. After the sample chamber was completely covered with dry-ice, the enclosure was opened and the DAC was tightly closed. Finally, the sample in the DAC was compressed to the target pressure of the experiment without intermediate heating.

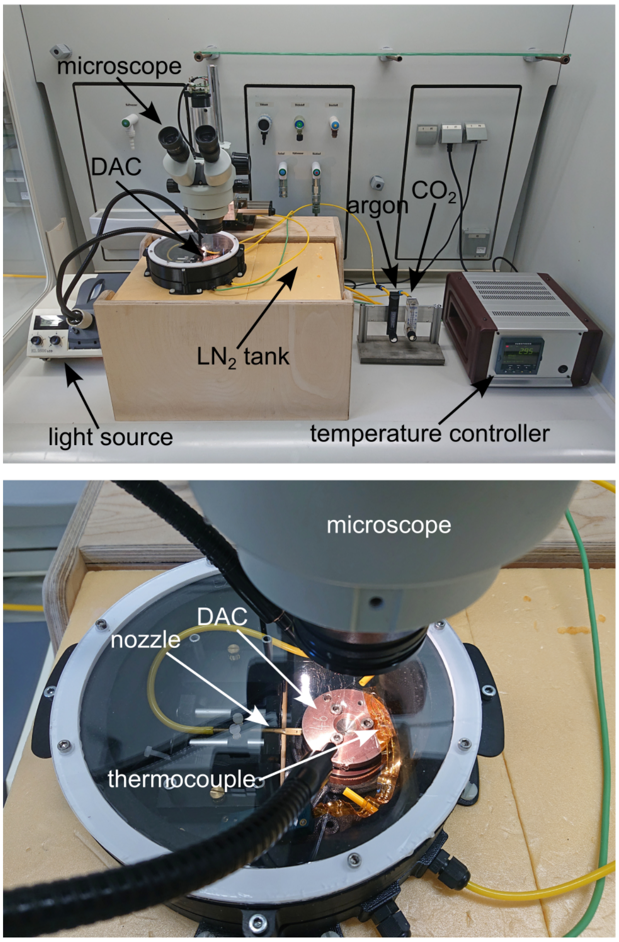

Figure S 1: Custom-built cryogenic DAC loading system with isolated liquid nitrogen (LN_2) storage tank, microscope, camera, nozzle, DAC, gas supply and temperature controller. The DAC is placed on the liquid nitrogen cooled Cu-holder.

*d.spahr@kristall.uni-frankfurt.de

1.4. Laser heating

The sample was laser-heated from both side using a custom-built set-up equipped with a Coherent Diamond K-250 pulsed CO_2 laser ($\lambda = 10600$ nm).[4] The laser power was adjusted to achieve a coupling of the laser to the sample, using a laser power of 1–6 W, resulting in a maximum temperature of $T_{\max} = 1600(200)$ K. The temperatures were determined by the two-color pyrometer method, employing Planck and Wien fits.[5] The heating time was $\approx$ 30 min. It is well established that laser-heating in DACs always suffers from large temperature gradients and the actual temperature is strongly dependent on the coupling of the laser with the sample, especially at lower temperatures. We estimate an uncertainty of at least $\pm$15% of the nominal temperature in the laser-heated region depending on the focus of the laser beam, based on typical 2D temperature-gradient determination experiments performed in DACs.[6]

1.5. Raman spectroscopy

Raman spectroscopy at high pressures was performed using an Oxford Instruments WITec alpha 300R Raman imaging microscope. The Raman microscope was equipped with an Olympus SLMPan N 50× objective. The measurements were performed with a 532 nm laser. We used the 1800 grooves mm^{-1} grating of the WITec UHTS 300S (VIS-NIR) spectrograph in combination with an Andor DR316B-LDC-DD CCD detector. The applied laser power was 100 mW on the sample and the spot size of the Raman laser was $\approx$ 0.8 µm. We assume a depth resolution of $\approx$ 6 µm in the direction of the laser beam. Raman maps were measured on a grid with a step-size of 1 µm. The background of the Raman spectra was corrected using the software package Fityk.[7]

1.6. Single crystal synchrotron X-ray diffraction

Single crystal synchrotron X-ray diffraction was carried out at the ESRF in Grenoble, France, at the high-pressure beam line ID27.[8] The beam size on the sample was 2×2 µm^2, focused by Kirkpatrick Baez mirrors. The diffraction data were collected using an Eiger2 X 9M CdTe detector, a wavelength of 0.3738 Å (33.2 keV) and a detector to sample distance of 184 mm. We rotated the DAC by $\pm 32^\circ$ around the vertical axis perpendicular to the beam while collecting frames in 0.5° steps with 2 s acquisition time per frame.

The detector to sample distance was calibrated using the powder diffraction pattern of a CeO_2 standard in conjunction with the software DIOPTAS.[9] The diffractometer/detector geometry for the analysis of the single crystal diffraction data was calibrated using diffraction data collected from a vanadinite ($Pb_5(VO_4)_3Cl$) single crystal in a DAC at ambient pressure. After the data collection, the reflections were indexed and integrated employing CrysAlis^{PRO} (version 43.67a).[10] We used the Domain Auto Finder program (DAFi) to find possible single crystal domains for the subsequent data reduction.[11] The structure solution and refinement were performed using the software package OLEX2 employing SHELXT for the crystal structure determination and SHELX for the refinement.[12,13,14]

1.7. Density functional theory-based calculations

First-principles calculations were carried out within the framework of density functional theory (DFT), employing the Perdew-Burke-Ernzerhof (PBE) exchange-correlation functional and the plane wave/pseudopotential approach implemented in the CASTEP simulation package.[15,16,17] "On the fly" norm-conserving or ultrasoft pseudopotentials generated using the descriptors in the CASTEP data base were employed in conjunction with plane waves up to a kinetic energy cutoff of 1020 eV or 630 eV, for norm-conserving and ultrasoft pseudopotentials, respectively. The accuracy of the pseudopotentials is well established.[18] A correction scheme for van der Waals (v.d.W.) interactions was applied in the DFT-calculations. We employed the correction scheme developed by Tkatchenko and Scheffler.[19] A Monkhorst-Pack grid was used for Brillouin zone integrations.[20] We used a distance between grid points of <0.023 Å^{-1}. Convergence criteria for geometry optimization included an energy change of $<5 \times 10^{-6}$ eV atom^{-1} between steps, a maximal force of <0.008 eV Å^{-1} and a maximal component of the stress tensor <0.02 GPa. Phonon frequencies were obtained from density functional perturbation theory (DFPT) calculations.[21,22] Raman intensities were computed using DFPT with the "$2n + 1$" theorem approach.[23]

All DFPT calculations were carried out with norm-conserving pseudopotentials. These are computationally more expensive than calculations with ultrasoft potentials, where the latter were employed for p, V-calculations. The differences in the results obtained with the two approaches is minor. Mulliken charges and bond populations calculations are carried out according to the formalism described by Segall *et al.*[24,25]. The projection of the plane wave states onto a localized basis uses a technique described by Sanchez-Portal *et al.*[26]. The population analysis of the resulting projected states uses the Mulliken formalism.[27]

2. Results

2.1. *Single crystal synchrotron X-ray diffraction at 30 GPa*

We performed synchrotron single-crystal X-ray diffraction on selected locations of the 2D grid across the sample chamber where unidentified reflections were present. Fig. S 2 a shows a part of an *unwarped* image of the raw-experimental data after processing of the ($hk\bar{1}$) area in CrysAlis, after the location of a suitable spot for the single crystal structure solution. Besides the reflection of the unknown carbonate phase, reflections and powder rings of different CO_2 phases (II, III, V), I_2O_5 and diamond are present in the diffraction data.

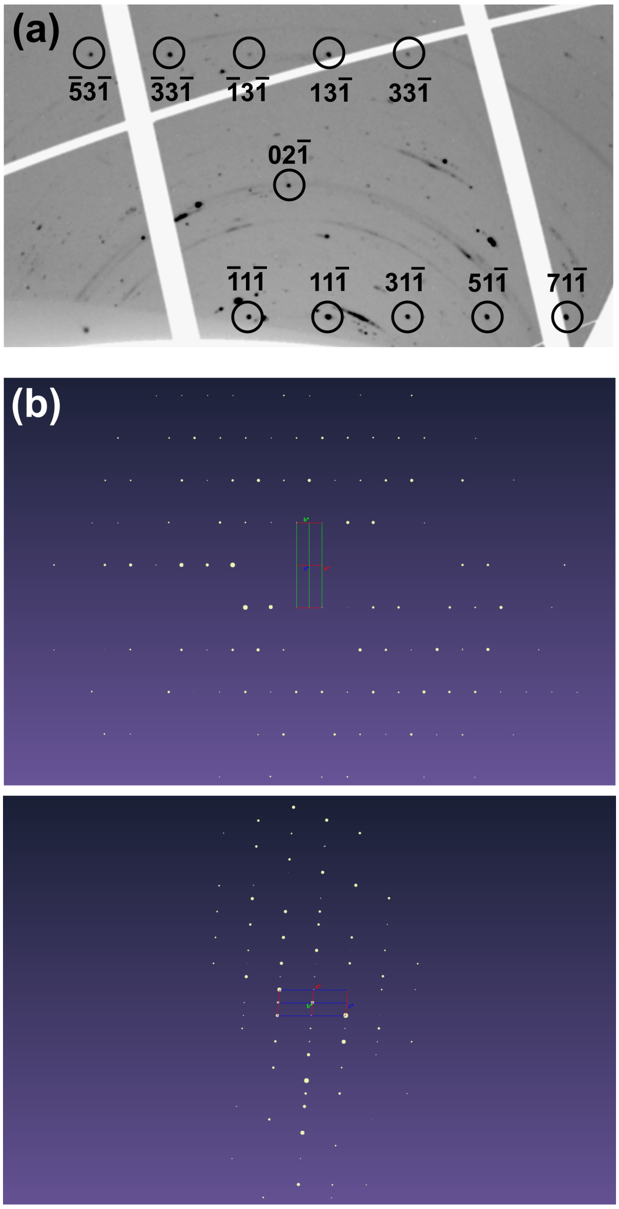

Figure S 2: (a) *Unwarped* image of the raw-experimental data after data reduction. The ($hk\bar{1}$) area is shown. Other reflections are due to other phases present in the sample chamber. (b) Schematic depiction of the reflections in reciprocal space using the Ewald-Explorer in CrysAlis after data reduction which were later used for the refinement. Projections of the reciprocal space are shown along c^* (top) and b^* (bottom).

Projections of the reciprocal space show the distribution of reflections and the effect of the shading of diffracted beams due to the metallic body of the DAC and the effect of the inactive detector-areas due to the segments of the Eiger2 X detector (Fig. S 2 b).

The crystal structure of the unknown carbonate phase in the monoclinic space group $C2/c$ (No. 15) with $Z = 4$ and a chemical composition of $(IO_2)_2[CO_3]$. The low R-value of 4.9% is indicative of a reasonable structure refinement. The displacement parameters of the heavy iodine atom was anisotropically, while the displacement parameters of the carbon and four oxygen atoms were refined isotropically. No constraints or restraints were applied for the refinement. The reflection to parameter ratio (11:1) is good for a DAC experiment. The crystallographic parameters of $(IO_2)_2[CO_3]$ at 30(2) GPa are listed in Table S 1 and in comparison to data derived from the DFT calculations.

Table S 1: Structural parameters of $(IO_2)_2[CO_3]$ at 30(2) GPa from single crystal structure solution (ambient temperature) in comparison to data from DFT calculations (athermal limit).

	**Single Crystal**	**DFT**
**Crystal data**		
Crystal system	monoclinic	monoclinic
Space group	$C2/c$	$C2/c$
Chemical formula	$(IO_2)_2[CO_3]$	$(IO_2)_2[CO_3]$
M_r	377.81	377.81
a (Å)	14.695(5)	14.847
b (Å)	4.429(1)	4.4577
c (Å)	5.67(1)	5.7304
α (°)	90.0	90.0
β (°)	94.30(8)	94.69
γ (°)	90.0	90.0
V (Å^3)	367.9(9)	377.98
Z	4	4
**Data collection**		-
F_{000}	672	-
θ range (°)	2.51–16.71	-
measured reflections	447	-
independent reflections	296	-
reflections $I > 2\sigma(I)$	170	-
R_{int}	0.034	-
		-
**Refinement**		-
$R[F^2 > 2\sigma(F^2)]$, $wR(F^2)$	0.049, 0.118	-
No. of reflections	296	-
No. of parameters	26	-
No. of restraints	0	-
No. of constraints	0	-
$\Delta\rho_{max}$, $\Delta\rho_{min}$ (e Å^{-3})	0.70, -0.91	-

By introducing a *rigu* constraint for the refinement of the displacement parameters, also the displacement parameters of the carbon and four oxygen atoms can be refined anisotropically. This results in a decrease of the R-value by $\approx 0.2\%$, but also in a significant decrease of the reflection to parameter ratio (6:1). Hence, we decided to use an isotropically refinement for the displacement parameters of the carbon and oxygen atoms.

2.2. *Bulk modulus of $(IO_2)_2[CO_3]$*

The bulk modulus (K_0) of $(IO_2)_2[CO_3]$ was derived from the p, V relation obtained from the DFT calculations. Our DFT-calculations were carried out between 0 GPa and 40 GPa. We fitted a 3rd-order Vinet equation of states (EoS) to unit cell volume obtained by the calculations (Fig. S 3) using the software package EOSFit7-GUI.[28,29] We used only the p, V data $\geq$ 10 GPa for the fit as the elastic stiffness tensor obtained at 0 GPa shows that at low pressure the structure is unstable against small deformations. The theoretical bulk modulus of $(IO_2)_2[CO_3]$ derived from the p, V relation is $K_0 = 15.2(6)$ GPa with $K_p = 9.33(7)$.

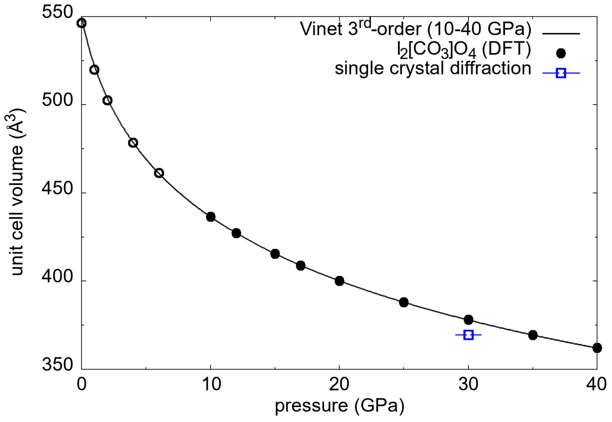

Figure S 3: A Vinet EoS was fitted to the unit cell volumes of $(IO_2)_2[CO_3]$ obtained by DFT-based calculations between 10–50 GPa. The open symbols were not used for the fit. The experimentally obtained unit cell volume from the single crystal structure solution at 30(2) GPa is shown for comparison.

Figure S 4 shows the relative changes in the lattice parameters of $(IO_2)_2[CO_3]$. The crystal structure is more compressible along the *b*-axis, while the compressibility along the *a*- and *c*-axes is significantly smaller.

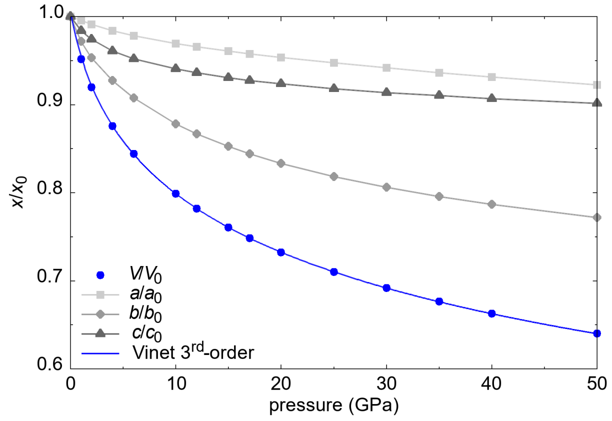

Figure S 4: Relative changes in the *a*, *b* and *c* lattice parameters of $(IO_2)_2[CO_3]$ derived from DFT-based calculations between 0 GPa and 50 GPa. The relative change in the unit cell volume together with the corresponding Vinet EoS fit (Fig. S 3) is shown for comparison.

2.3. *Phonon dispersion of $(IO_2)_2[CO_3]$*

We employed the DFT calculations to derive the phonon dispersion curves for $(IO_2)_2[CO_3]$ at 30 GPa (Fig. S 5). The phonon dispersion curves show that $(IO_2)_2[CO_3]$ is dynamically stable at this pressure.

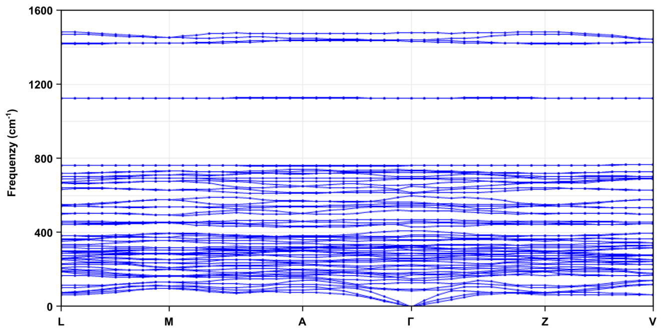

Figure S 5: Phonon dispersion curves for $(IO_2)_2[CO_3]$ at 30 GPa from DFT-based calculations.

References

(1) Boehler, R. New diamond cell for single–crystal X-ray diffraction. *Rev. Sci. Instrum.* **2006**, *77*, 115103–1–115103–3, DOI: 10.1029/JB091iB05p04673

(2) Mao, H. K.; Xu, J.; Bell, P. M. Calibration of the ruby pressure gauge to 800 kbar under quasi-hydrostatic conditions. *J. Geophys. Res.* **1986**, *91*, 4673–4676, DOI: 10.1029/JB091iB05p04673

(3) Spahr, D.; König, J.; Bayarjargal, L.; Luchitskaia, R.; Milman, V.; Perlov, A.; Liermann, H.-P.; Winkler, B.; Synthesis and Structure of Pb[C_2O_5]: An Inorganic Pyrocarbonate Salt. *Inorg. Chem.* **2022**, *61*, 9855–9859, DOI: 10.1021/acs.inorgchem.2c01507

(4) Bayarjargal, L.; Fruhner, C.-J.; Schrodt, N.; Winkler, B. $CaCO_3$ phase diagram studied with Raman spectroscopy at pressures up to 50 GPa and high temperatures and DFT modeling. *Phys. Earth Planet. Inter.* **2018**, *281*, 31–45, DOI: 10.1016/j.pepi.2018.05.002

(5) Benedetti, L. R.; Loubeyre, P. Temperature gradients, wavelength-dependent emissivity, and accuracy of high and very-high temperatures measured in the laser-heated diamond cell. *High Press. Res.* **2004**, *24*, 423–455, DOI: 10.1080/08957950412331331718

(6) Du, Z.; Amulele, G.; Benedetti, L. R.; Lee, K. K. M. Mapping temperatures and temperature gradients during flash heating in a diamond-anvil cell. *Rev. Sci. Instrum.* **2013**, *84*, 075111, DOI: 10.1063/1.4813704

(7) Wojdyr, M. *Fityk*: a general-purpose peak fitting program. *J. Appl. Cryst.* **2010**, *43*, 1126–1128, DOI: 10.1107/S0021889810030499

(8) Mezouar, M.; Garbarino, G.; Bauchau, S.; Morgenroth, W.; Martel, K.; Petitdemange, S.; Got, P.; Clavel, C.; Moyne, A.; Van Der Kleij, H.-P.; Pakhomova, A.; Wehinger, B.; Gerin, M.; Poreba, T.; Rosa, A.; Forestier, A.; Weck, G.; Datchi, F.; Wilke, M.; Jahn, S.; Andrault, D.; Libon, L.; Pennacchioni, L.; Laniel, D.; Bureau H. The high flux nano-X-ray diffraction, fluorescence and imaging beamline ID27 for science under extreme conditions on the ESRF Extremely Brilliant Source. *High Press. Res.* **2024**, *44*, 171–198, DOI: 10.1080/08957959.2024.2363932

(9) Prescher, C.; Prakapenka, V. B. *DIOPTAS*: a program for reduction of two-dimensional X-ray diffraction data and data exploration. *High. Press. Res.* **2015**, *35*, 223–230, DOI: 10.1080/08957959.2015.1059835

(10) Agilent, CrysAlis PRO, Yarnton, England, **2014**

(11) Aslandukov, A.; Aslandukov, M.; Dubrovinskaia, N.; Dubrovinsky, L. *Domain Auto Finder* (*DAFi*) program: the analysis of single-crystal X-ray diffraction data from polycrystalline sample. *J. Appl. Cryst.* **2022**, *55*, 1383–1391, DOI: 10.1107/S1600576722008081

(12) Dolomanov, O. V.; Bourhis, L. J.; Gildea, R. J.; Howard, J. A. K.; Puschmann, H. *OLEX2*: a complete structure solution, refinement and analysis program. *J. Appl. Cryst.* **2009**, *42*, 339–341, DOI: 10.1107/S0021889808042726

(13) Sheldrick, G. M. *SHELXT* — Integrated space-group and crystal-structure determination. *Acta. Cryst.* **2015**, *A71*, 3–8, DOI: 10.1107/S2053273314026370

(14) Sheldrick, G. M. Crystal structure refinement with *SHELXL*. *Acta. Cryst.* **2015**, *C71*, 3–8, DOI: 10.1107/S2053229614024218

(15) Hohenberg, P.; Kohn, W. Inhomogeneous Electron Gas. *Phys. Rev.* **1967**, *136*, B864–B871, DOI: 10.1103/PhysRev.136.B864

(16) Perdew, J. P.; Burke, K.; Ernzerhof, M. Generalized Gradient Approximation Made Simple. *Phys. Rev. Lett.* **1996**, *77*, 3865–3868, DOI: 10.1103/PhysRevLett.77.3865

(17) Clark, S. J.; Segall, M. D.; Pickard, C. J.; Hasnip, P. J.; Probert, M. I. J.; Refson, K.; Payne, M. C. First principles methods using CASTEP. *Z. Kristallogr.* **2005**, *220*, 567–570, DOI: 10.1524/zkri.220.5.567.65075

(18) Lejaeghere, K.; Bihlmayer, G.; Björkman, T.; Blaha, P.; Blügel, S.; Blum, V.; Caliste, D.; Castelli, I. E.; Clark, S. J.; Dal Corso, A. et al. Reproducibility in density functional theory calculations of solids. *Science* **2016**, *351*, aad3000, DOI: 10.1126/science.aad3000

(19) Tkatchenko, A.; Scheffler, M. Accurate Molecular Van Der Waals Interactions from Ground-State Electron Density and Free-Atom Reference Data. *Phys. Rev. Lett.* **2009**, *102*, 073005, DOI: 10.1103/PhysRevLett.102.073005

(20) Monkhorst, H. J.; Pack, J. D. Special points for Brillouin-zone integrations. *Phys. Rev. B* **1976**, *13*, 5188–5192, DOI: 10.1103/PhysRevB.13.5188

(21) Baroni, S.; de Gironcoli, S.; Dal Corso, A.; Giannozzi, P. Phonons and related crystal properties from density-functional perturbation theory. *Rev. Mod. Phys.* **2001**, *73*, 515–562, DOI: 10.1103/RevModPhys.73.515

(22) Refson, K.; Tulip, P. R.; Clark, S. J. Variational density-functional perturbation theory for dielectrics and lattice dynamics. *Phys. Rev. B* **2006**, *73*, 155114, DOI: 10.1103/PhysRevB.73.155114

(23) Miwa, K. Prediction of Raman spectra with ultrasoft pseudopotentials. *Phys. Rev. B* **2011**, *84*, 094304, DOI: 10.1103/PhysRevB.84.094304

(24) Segall, M. D.; Pickard, C. J.; Shah, R.; Payne, M. C. Population analysis in plane wave electronic structure calculations. *Mol. Phys.* **1996**, *89*, 571–577, DOI: 10.1080/002689796173912

(25) Segall, M. D.; Shah, R.; Pickard, C. J.; Payne, M. C. Population analysis of plane-wave electronic structure calculations of bulk materials. *Phys. Rev. B* **1996**, *54*, 16317–16320, DOI: 10.1103/PhysRevB.54.16317

(26) Sanchez-Portal, D.; Artacho, E.; Soler, J. M. Projection of plane-wave calculations into atomic orbitals. *Solid State Commun.* **1995**, *95*, 685–690, DOI: 10.1016/0038-1098(95)00341-X

(27) Mulliken, R. S. J. Electronic Population Analysis on LCAO–MO Molecular Wave Functions. *J. Chem. Phys.* **1955**, *23*, 1833–1846, DOI: 10.1063/1.1740588

(28) Vinet, P.; Rose, J. H.; Ferrante, J.; Smith, J. R. Universal features of the equation of state of solids. *Phys.: Condens. Matter* **1989**, *1*, 1941–1963, DOI: 10.1088/0953-8984/1/11/002

(29) Gonzalez-Platas, J.; Alvaro, M.; Nestola, F.; Angel, R. *EosFit7-GUI*: a new graphical user interface for equation of state calculations, analyses and teaching. *J. Appl. Cryst.* **2016**, *49*, 1377–1382, DOI: 10.1107/S1600576716008050
